# Gabi wheat a panel of European elite lines as central stock for wheat genetic research

**DOI:** 10.1038/s41597-022-01651-5

**Published:** 2022-09-02

**Authors:** Abhishek Gogna, Albert W. Schulthess, Marion S. Röder, Martin W. Ganal, Jochen C. Reif

**Affiliations:** 1 Leibniz Institute of Plant Genetics and Crop Plant Research (IPK), 06466 Stadt Seeland, Germany; 2 SGS Institut Fresenius GmbH, TraitGenetics Section, Am Schwabeplan 1b, 06466, Stadt Seeland OT Gatersleben, Germany

**Keywords:** Plant breeding, Agricultural genetics

## Abstract

In plant sciences, curation and availability of interoperable phenotypic and genomic data is still in its infancy and represents an obstacle to rapid scientific discoveries in this field. To that end, supplementing the efforts being made to generate open access wheat genome, pan wheat genome and other bioinformatic resources, we present the GABI-WHEAT panel of elite European cultivars comprising 358 winter and 14 summer wheat varieties released between 1975 to 2007. The panel has been genotyped with SNP arrays of increasing density to investigate several important agronomic, quality and disease resistance traits. The robustness of investigated traits and interoperability of genomic and phenotypic data was assessed in the current publication with the aim to transform this panel into a public data resource for future genetic research in wheat. Consecutively, the phenotypic data was formatted to comply with FAIR principles and linked to online databases to substantiate panel origin information and quality. Thus, we were able to make a valuable resource available for plant science in a sustainable way.

## Abstract (summary)

**Table:** 

Measurement(s)	agronomic, quality and disease traits
Technology Type(s)	manual measurement in the field
Sample Characteristic - Organism	Triticum aestivum L.

## Background & Summary

The research landscape for wheat (*Triticum aestivum* L.) is witness to unprecedented developments owing to the availability of multi-omics data and advances in breeding informatics. These developments fueled the discoveries of marker-trait associations, gene cloning(s)^1^, targeted gene editing(s), and better understanding into genetic architecture of complex traits. However, the upcoming decade poses new challenges in the face of climate change, evolving consumer food preferences and sociopolitical scenarios between the wheat producing and importing countries. This implies that the conventional wheel of research output has to now turn even faster without compromising on quality and throughput. Current crop growth models driven by climate change scenarios already predict forthcoming changes in temperature, rainfall and spatiotemporal alterations in pathogen pressures across Europe, which if left unchecked, could lead to dwindling yields and massive crop loss^2^.

In the past, whereas the genetic mapping for important traits benefitted from availability of high-density markers in the form of SNP arrays and even better from whole genome sequencing, modern research requirements necessitate a look beyond the now saturated genomic data generation technologies. Availability and choice of a genetically diverse panel with robust phenotypic data is, therefore, crucial. Several multi-parental populations covering a wide spectrum of traits for major crops like maize, barley, rapeseed, rice, soybean, cotton including wheat now exist^3^ and aim to address this issue. But the main limitation of such populations is that the genetic diversity space is defined by the founders/parents. Obviously, this limited number would hardly cover the genetic diversity existent in the elite pool for the crop. Elite breeding lines in Europe, culminated from years of commercial development, are a precise snapshot of region-specific variation required for optimal trait expression. As such these form an excellent open-ended core resource for genetic studies that can be extended with latest released cultivars.

A European panel of elite winter as well as some summer wheat cultivars, denoted as GABI-WHEAT, assembled from varieties released between 1975 to 2007, representing almost four decades of breeding efforts in European wheat breeding companies was curated in 2013^4^ and has since been used extensively for major developments in wheat across Europe. The panel was initially genotyped with SSR markers^4^, but given the popularity of the panel, over the years the genotypes therein were typed with SNP marker arrays with increasing marker densities viz. 35k^5^, 90k^6^, and 135k^7^ with the aim to expand the canvas for novel association discovery.

Studies have benefitted from this expansion and have reported novel associations for previously reported disease^8,9^, and quality traits^7^. Exploiting the substantial genetic diversity existing in GABI-WHEAT panel for lipid activity^10^, efforts have been made to develop metabolomic methods for quantifying oxidative stability of lipid oxidases^11^ and to hasten development of lipid stable wheat varieties for diverse markets^12^. Beyond that, high-throughput phenotyping methods have been developed using GABI-WHEAT panel to augment genetic variant discovery using multi sensor field phenotyping platform^13^, hyperspectral canopy sensing^14^ as well as multi-image unmanned aerial vehicle based field phenotyping^15^ for stem elongation, *Septoria tritici* blotch, and for measuring plot canopy temperatures. Additionally, the panel has been used to study plant pathogen interactions and propose mechanism of possible tradeoff between tolerance and resistance in elite wheat cultivar for *Septoria tritici* blotch^16^. Nevertheless, these developments are still in infancy and for limited traits. At the same time, high-throughput phenotyping is constantly expanding the array of traits to study involving for example root phenotyping. If near-term trends are even marginally indicative, then open sharing of proven and robust panels like GABI-WHEAT could not only cut costs in future developments but also save crucial research time needed for data generation.

It is reasonable to expect that to support population pressure by 2050, crop production must rise and this would be possible given high throughput quality research. In line with developing public access resources to enable next generations of scientists spend less time on generating data and more time working with as well as building upon curated data, we publish herein the GABI-WHEAT panel including the original phenotypic data^4^ and recently generated marker data^5,7^ as well as respective marker oligo sequences. Our contribution to the scientific community is a step to (1) augment the wheat research landscape in Europe for fundamental research topics, (2) hasten the translation of scientific learnings into elite variety development, and (3) promote further resource development and sharing.

## Methods

### Phenotypic data

The phenotypic data corresponds to seven agronomic [heading date (**HD)**, plant height (**PH)**, thousand grain weight (**TGW)**, ear weight (**EW)**, grains per ear (**GPE**), yield (**YIE**), specific weight (**SW**)], six quality [grain hardiness (**GH)**, starch content (**STC**), protein content (**PC**), sedimentation index (**SDS**), Hagberg falling number (**HAG**), zeleny sedimentation index (**ZEL**)] and three disease [resistance to fusarium head blight (**FHB)**, resistance to septoria blotch (**STB**), existence to tan spot (**DTR**)] traits for GABI-WHEAT panel comprising 358 winter and 14 spring wheat varieties. For the field trials nine checks were added in >1 replications to round total number of genotypes per trial to 400^4^. Curation of phenotypic data for agronomic and quality traits was done from field experiments randomized according to alpha-lattice designs with two replications. These trials were conducted at up to 5 locations (Andelu/France; Seligenstadt/Germany; Wohlde/Germany; Janville/France; Saultain/France) in up to two years (2009; 2010). Investigations of disease resistance traits were done on randomized complete block design with three replications per site at up to 4 locations (Ahlum/Germany; Lafferde/Germany; Cecilienkoog/Germany; Halle-Bodenwerder/Germany) in up to two years (2009; 2010). Each year and location combination were considered as one environment. The grain moisture content for measurement of traits was standardized to 14%.

### Genomic data

The genomic data used herein derives from three different marker platforms, viz. 35k Affymetrix^5,8^, 90k iSELECT^6^ and 135k^7^ SNP arrays for 371, 372, and 186 genotypes (GABI-WHEAT-TROST panel) respectively, out of the total 372 individuals. The number of markers remaining after quality check including filtering of markers with more than five percent heterozygous calls, missing values as well as minor allele frequency were 9,494, 18,776, and 35,258 respectively. Imputation of missing values in the filtered marker datasets was done using Random Forest regression^17,18^.

### Phenotypic data analysis

**Fig. 1:**
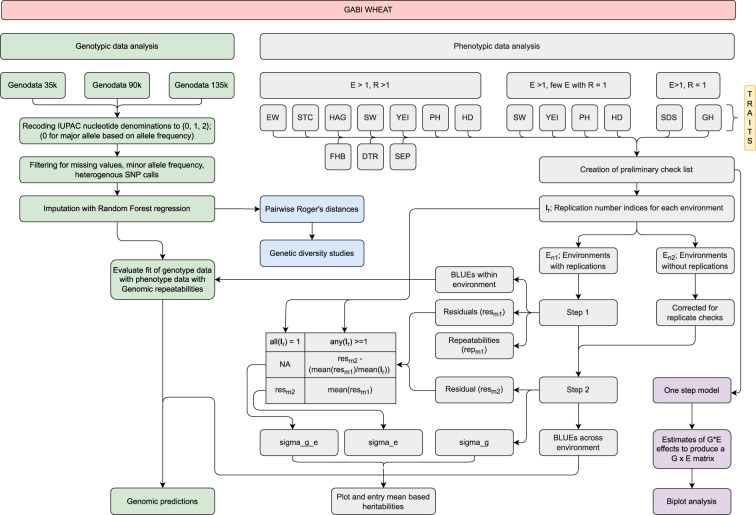
Overview of the analysis.

An unweighted two-stage univariate^19^ mixed model analysis was adopted to analyze the phenotypic traits (Fig. 1). In the first step, best linear unbiased estimates (BLUEs) were derived per environment for each trait with the following model:

1 $${{\rm{y}}}_{{\rm{ijk}}}={\rm{\mu }}+{{\rm{g}}}_{{\rm{i}}}+{{\rm{r}}}_{{\rm{j}}}+{{\rm{b}}}_{{\rm{k}}}\left({{\rm{r}}}_{{\rm{j}}}\right)+{{\rm{e}}}_{{\rm{ijk}}}{\rm{,}}$$

where, y_ijk_ denotes trait measurement from i^th^ genotype (g) in k^th^ block (b) nested in j^th^ replication (r). In the model (1) all terms except the common mean (µ) and g_i_ were considered random for deriving BLUEs, whereas all terms except µ were modelled as random to estimate variance(s) for deriving repeatabilities per environment as,

2 $${{\rm{R}}}_{{\rm{n}}}={{\rm{\sigma }}}_{{\rm{g}}}^{2}/\left({{\rm{\sigma }}}_{{\rm{g}}}^{2}+{{\rm{\sigma }}}_{{\rm{g}}}^{2}/{{\rm{n}}}_{{\rm{r}}}\right),$$

where, R_n_ denotes repeatability for a trait at n^th^ environment, σ^2^_g_ denotes the genotypic variance, σ^2^_e_ denotes the error variance and n_r_ denotes number of replications. In the second step, BLUEs across environments were calculated with the model,

3 $${{\rm{y}}}_{{\rm{im}}}={\rm{\mu }}+{{\rm{g}}}_{{\rm{i}}}+{{\rm{E}}}_{{\rm{m}}}+{{\rm{e}}}_{{\rm{im}}},$$

where, y_im_ denotes trait measurement from i^th^ genotype in the m^th^ environment (E). In the model (3) all except the µ and g_i_ terms were considered random for deriving BLUEs, whereas all terms except µ were modelled as random to estimate variance(s) for deriving heritabilities for a given trait. The normality of the distribution of BLUEs across environments was tested for each trait with the Shapiro–Wilk test at p = 0.05. Heritability was estimated as:

4 $${{\rm{h}}}_{{\rm{plotbased}}}^{2}={{\rm{\sigma }}}_{g}^{2}/\left({{\rm{\sigma }}}_{g}^{2}+{{\rm{\sigma }}}_{{g}^{* }{\rm{e}}}^{2}+{{\rm{\sigma }}}_{g}^{2}\right),$$

and

5 $${{\rm{h}}}_{{\rm{entry}}\;{\rm{mean}}\;{\rm{based}}}^{2}={\sigma }_{{\rm{g}}}^{2}/\left({\sigma }_{{\rm{g}}}^{2}+{\sigma }_{{{\rm{g}}}^{* }{\rm{e}}}^{2}/\left({{\rm{n}}}_{{\rm{e}}}\right)+{\sigma }_{{\rm{e}}}^{2}/\left({{\rm{n}}}_{{\rm{e}}}^{\ast }{{\rm{n}}}_{{\rm{r}}}\right)\right){\rm{,}}$$

where, n_e_ denotes number of environments and n_r_ stands for (mean) number of replications.

For disease resistance traits (FHB, SEP, DTR), since complete-block structure was missing the previously described model (1) is reduced to:

6 $${{\rm{y}}}_{{\rm{ij}}}={\rm{\mu }}+{{\rm{g}}}_{{\rm{i}}}+{{\rm{r}}}_{{\rm{j}}}+{{\rm{e}}}_{{\rm{ij}}},$$

For the traits having few environments with just one replication (TKW, PC, ZEL, GPE) or traits with no complete-block replication in any of the environment (SDS, GH), the previously described model (1) was modified as follows,

7 $${{\rm{y}}}_{{\rm{ik}}}={\rm{\mu }}+{{\rm{g}}}_{{\rm{i}}}+{{\rm{b}}}_{{\rm{k}}}+{{\rm{e}}}_{{\rm{ik}}},$$

For disease resistance traits mean values for genotypes in a given replication of the respective trial were calculated (1) First across two assessments and then over two types of leaves for DTR as well as STB; (2) Across three assessments separately for incidence and severity score for FHB. In the latter case, an FHB score was additionally calculated as,

8 $$\left({\rm{mean}}\;{\rm{incidence}}\;{\rm{score}}\;{\rm{across}}\;{\rm{three}}\;{\rm{assessments}}\right)\times \left({\rm{mean}}\;{\rm{severity}}\;{\rm{score}}\;{\rm{across}}\;{\rm{three}}\;{\rm{assessments}}\right)\,/\,100$$

### Biplot analysis

The genotype times environment (random) effects matrix (g*e matrix) was derived by fitting a one-step model, i.e.,

9 $${{\rm{y}}}_{{\rm{ijkm}}}={\rm{\mu }}+{{\rm{g}}}_{{\rm{i}}}+{{\rm{E}}}_{{\rm{m}}}+{{\rm{g}}}_{{\rm{i}}}:{{\rm{E}}}_{{\rm{m}}}+{{\rm{r}}}_{{\rm{j}}}+{{\rm{b}}}_{{\rm{k}}}+{{\rm{e}}}_{{\rm{ijkm}}},$$

for agronomic as well as quality traits, and

10 $${{\rm{y}}}_{{\rm{ijm}}}={\rm{\mu }}+{{\rm{g}}}_{{\rm{i}}}+{{\rm{E}}}_{{\rm{m}}}+{{\rm{g}}}_{{\rm{i}}}:{{\rm{E}}}_{{\rm{m}}}+{{\rm{r}}}_{{\rm{j}}}+{{\rm{e}}}_{{\rm{ijm}}},$$

for disease traits respectively. In model (9) as well as (10), all components except µ were assumed random and the biplot was produced from a rank two approximation of the centered g*e matrix as outlined in^20^.

### Genomic-phenomic data interoperaty

Genomic repeatability was used as a measure of data interoperability and was calculated for BLUEs within each environment with the three types of marker datasets by simultaneously modelling additive and additive*additive epistasis^21^ using the following model,

11 $${\rm{y}}={1}_{{\rm{n}}}{\rm{\mu }}+{{\rm{g}}}_{1}+{{\rm{g}}}_{2}+{\rm{e,}}$$

where, y denotes an n-dimensional vector of phenotypic records, 1_n_ denotes an n-length vector of ones, µ stands for the population mean of the trait under investigation, g_1_ and g_2_ denote additive and additive*additive epistatic genotypic values respectively. µ was assumed fixed, whilst g_1_ ~ N(0, G*σ^2^_g1_), g_2_ ~ N(0, H*σ^2^_g2_) and e ~ N(0, I*σ^2^_e_). G was an n × n genomic relationship matrix calculated following^22^ and H was subsequently calculated as the Hadamard product of G with itself. In the model (8) it was assumed that cov (g_1_, g_2_) = cov (g_1_, e) = cov (g_2_, e) = 0. The model (9) was implemented with BGLR^23^ inside R^24^ with an *apriori* kernel set to “RKHS” for both kinship matrices.

Genomic repeatability was thereafter defined in two ways as (1) narrow-sense genomic repeatability (R_n_) and (2) broad-sense genomic repeatability (R_b_):

12 $${{\rm{R}}}_{{\rm{n}}}={\sigma }_{{\rm{g1}}}^{2}/\left({\sigma }_{{\rm{g}}1}^{2}+{\sigma }_{{\rm{g}}2}^{2}+{\sigma }_{{\rm{e}}}^{2}\right){\rm{\& }}$$

13 $${{\rm{R}}}_{{\rm{b}}}={\sigma }_{{\rm{g1}}}^{2}+{\sigma }_{{\rm{g2}}}^{2}/\left({\sigma }_{{\rm{g}}1}^{2}+{\sigma }_{{\rm{g2}}}^{2}+{\sigma }_{{\rm{e}}}^{2}\right){\rm{,}}$$

Lastly, genomic predictions for BLUEs across environments was calculated using a 5-fold cross validation implemented 100 times with the model (11), separately for each source of genotypic data. Genomic prediction ability was thereafter defined as the correlation between BLUEs across environments for a trait and those predicted with model (11).

## Data Records

The phenotypic data produced herein is formatted in ISA-TAB format^25^ to enable FAIR use by diverse audience engaged in wheat research landscape. The description of the experiments including metadata adheres to the standards defined by MIAPPE 1.1^26^. The phenotypic data correspond to seven agronomic (HD, PH, TGW, EW, GPE, YIE, SW), six quality (GH, STC, PC, SDS, HAG, ZEL), and three disease resistance traits (FHB, STB, DTR) for the GABI-WHEAT panel. The entire array of genotypic data(s) and marker oligo sequences with different marker densities for GABI-WHEAT as well as for GABI-WHEAT-TROST panel (a subset of GABI-WHEAT panel) is also being published. The phenotypic data for this publication is available at e!DAL-PGP-Repository^27^ and the genotypic data along with marker oligo sequences is accessible at dryad repository^28^.

The varieties analyzed herein originate from over 12 European countries wherein they were first registered in the period 1975 to 2007^29^. Originally, the observations were made for agronomic, quality and disease traits in 2009 and 2010. However, for the purpose of current publication the original data was reformatted into ISA-TAB format. It includes an investigation file outlining the general features of the original data as well as study and assay files for each experimental design. Each pair (study + assay file) corresponds to data collected in a given experimental design viz. alpha-lattice design (for agronomic and quality traits) and randomized complete block design (for disease traits). The study file describes the genotypes analyzed in the respective trial design, specifically it has information on (1.) Organism studied (Characteristics[Organism]), (2.) Name of reference database for the organism [Term Source REF], (3.) Access information of the organism in reference database [Term Accession Number], (4.) Variety evaluated (Characteristics[Variety Name]), (5.) Access information to the variety in the public database [Variety Database 1 (http://wheatpedigree.net/) and 2 (https://www.proplanta.de/)], (6.) Design effects (Factor Value[…]), (7.) Mapping of study file rows to assay file (Sample Name), (8.) Coding used to resolve design effects and connecting to genodata (Characteristics [Original Coding]) and other information like location coordinates. The assay file on the other hand records the traits phenotyped in given environment(s) (Harvest year + Location), specifically each row in the assay file connects via ‘Sample Name’, the relevant rows of study file to measurements for phenotypic/quality or disease traits in the assay file.

Phenotypic data for agronomic and disease traits were recorded across two seasons (in two years) at up to 5 locations in Germany or France. In Germany, respective locations i.e. Wohlde and Seligenstadt were available in both seasons, whereas in France for season one of the two locations was unavailable due to slug damage. So, to compensate for the loss of a location in season one, three locations (Andelu, Janville, Saultain) instead of two were used for phenotypic evaluation in season two. Phenotypic data for all 8 environments was available for HD, PH, TKW, YIE, PC and ZEL. The data for EW, STC as well as HAG was only available for German environments, whilst that for GH and SDS was only available for French environments. The data for the two remaining traits viz, SW and GPE was available only for few environments in both Germany and France (Table 1).

**Table 1:** Description of trial structure for various traits in GABI-WHEAT.

Traits	Abbreviations	Locations in 2009			Locations in 2010				
Heading date	HD	Andelu (2)	Seligenstadt (2)	Wohlde (2)	Andelu (2)	Janville (2)	Seligenstadt (2)	Wohlde (2)	Saultain (2)
Plant height	PH	Andelu (2)	Seligenstadt (2)	Wohlde (2)	Andelu (2)	Janville (2)	Seligenstadt (2)	Wohlde (2)	Saultain (2)
Thousand grain weight	TKW/TGW	Andelu (2)	Seligenstadt (2)	Wohlde (2)	Andelu (1)	Janville (1)	Seligenstadt (1)	Wohlde (2)	Saultain (2)
Ear weight	EW			Wohlde (2)				Wohlde (2)	
Grains per ear	GPE	Andelu (1)		Wohlde (2)	Andelu (2)				
Yield	YIE	Andelu (2)	Seligenstadt (2)	Wohlde (2)	Andelu (2)	Janville (2)	Seligenstadt (2)	Wohlde (2)	Saultain (2)
Specific weight	SW	Andelu (2)	Seligenstadt (2)	Wohlde (2)			Seligenstadt (2)	Wohlde (2)	
Grain hardiness	GH	Andelu (1)			Andelu (1)	Janville (1)			Saultain (1)
Starch content	STC/GSC		Seligenstadt (2)	Wohlde (2)				Wohlde (2)	
Protein content	PC/GPC	Andelu (1)	Seligenstadt (2)	Wohlde (2)	Andelu (1)	Janville (1)	Seligenstadt (2)	Wohlde (2)	Saultain (1)
Sedimentation index	SDS	Andelu (1)			Andelu (1)	Janville (1)			Saultain (1)
Hagberg falling number	HAG		Seligenstadt (2)	Wohlde (2)			Seligenstadt (2)	Wohlde (2)	
Zeleny sedimentation index	ZEL	Andelu (1)	Seligenstadt (2)	Wohlde (2)	Andelu (1)	Janville (1)	Seligenstadt (2)	Wohlde (2)	Saultain (1)
Resistance to fusarium head blight	FHB	Cecilienkoog (3)	Ahlum (3)		Halle-Bodenwerder (3)	Ahlum (3)			
Resistance to septoria blotch	STB	Cecilienkoog (3)			Cecilienkoog (3)				
Resistance to tan spot	DTR				Lafferde (3)	Ahlum (3)			

Location coordinates – *Andelu* - 48.8°N 1.8°E; height = 120.8 m, *Seligenstadt* - 50.0°N 8.9°E; height = 113.4 m, *Wohlde* - 54.4°N 9.2°E; height = 11.6 m, *Janville* - 48.2°N 1.8°E; height = 135.0 m, *Saultain* - 50.3°N 3.5°E; height = 72.5 m, *Cecilienkoog* - 54.5°N 8.9°E; height = 0.7 m, *Ahlum* - 52.6°N 11.0°E; height = 45.4 m, *Halle-Bodenwerder* - 51.9°N 9.5°E; height = 98.4 m, *Lafferde* - 52.2°N 10.2°E; height = 87.4 m. Values in brackets ({1–3}) denote the number of replications available at that location for a given trait and environement.

Phenotypic data for disease traits was collected from separate inoculation trials at different locations in Germany albeit in the same two seasons. The phenotypic data for FHB was available for Ahlum and Cecilienkoog for season one and for Ahlum and Halle‐Bodenwerder for season two. The phenotypic data for STB was available only for Cecilienkoog for both seasons, whilst that of DTR was available only for season two at two locations viz. Ahlum and Lafferde (Table 1). The curation of each trait along with other relevant data is summarized below.

### Agronomic Traits

#### Heading date (HD)

Total days from the 1^st^ of January, when approximately half of the ears per plot were fully visible i.e. at BBCH 59 from the Zadoks growth scale^30,31^.

#### Plant height (PH)

Average plant height per plot was measured before harvest, in centimeters, without awns^32^.

#### Thousand grain weight (TGW)

For French environments; 500 grains were counted with a mechanical counter “Contador” and weighted. For German environments; grains in 10 g sample per plot were counted using the mechanical counter “Pfeuffer Contador”. Finally, all weight/grain values were extrapolated to 1000 grains^33^ and expressed in grams.

#### Ear weight (EW)

Average of 10 ear sample weights per plot. Ear samples were taken before harvest and expressed in grams^34^.

#### Grains per ear (GPE)

Average number of grains per ear from 10 ear samples per plot. Ear samples were taken before harvest^34^.

#### Yield (YIE)

Plot yield after combine harvest was extrapolated to an area of one hectare and expressed in quintal per hectare^34^.

#### Specific weight (SW)

A 250-milliliter cylinder was filled up to the top with a clean grain sample from each harvested plot. The weight/volume value of the sample was extrapolated to 100 liters and expressed in kilogram/hectoliter^34^.

### Quality traits

#### Grain hardiness (GH), starch content (STC), and protein content (PC)

A 400 g grains sample per harvested plot was analyzed using an OmegAnalyzer G (Bruins Instruments) with wavelengths of 730–1100 nm. Observations were recorded in percentages^7^.

#### Sedimentation test (SDS)

Eight grain samples per plot, were ground and mixed at rate 6.3 g per sample to 50 ml of distilled water taken in 100 ml graduated cylinder. After proper mixing and shaking, mean sedimentation values were recorded across the eight samples with a 0.5 ml precision. Values were adjusted according to the temperature of sedimentation liquid using AACC standardization tables^35^.

#### Hagberg falling number (HAG)

A 250 g of representative seed sample per plot was ground, from which 7 g flour was added to a dry falling number tube and suspended by mixing with 25 ml distilled water at 22 ± 2 °C. Viscometer was then inserted and the combination was immediately (30–60 seconds of mixing) placed in water bath. The timer was started simultaneously. After the viscometer falls the standard threshold distance, the end time was recorded in seconds. Difference between start and end time was reported as falling number^36^.

#### Zeleny sedimentation index (ZEL)

Four-gram grain sample was ground, sieved and 0.32 g of flour was taken in a 10 ml stoppered graduated cylinder. Five ml of bromophenol blue solution was added to the cylinder. After proper mixing, 5 ml of lactic acid reagent was added and mixing was done again. Cylinder was then put on a stand and sedimentation volume was recorded with a 0.01 ml precision. The obtained micro sedimentation values were transformed to macro sedimentation values using AACC standardization tables^37^.

### Disease traits

#### Resistance to Fusarium head blight (FHB)

Spray inoculations were done with 50,000 spores per ml using a 1:2 mixture of *F. graminearum* and *F. culmorum* isolates, respectively, with water volume of 600 L/ha. Three inoculations were done at 10 days interval starting at BBCH 61. Incidence and severity were recorded in 3 assessments 20, 28 and 33 days after the first inoculation. Incidence was visually rated as percentage of infected spikes from 50 infected spikes per plot, whereas severity was visually rated as the percentage of infected area per spike of the infected spikes^4^. Low values indicate low infection and vice versa.

#### Resistance to Septoria blotch (STB)

Spray inoculations were done with 5 × 10^6^ spores per ml of pycnidiospores using a water volume of 600 L/ha. Two inoculations were done at 10 days interval starting from BBCH 39/41. To augment infection risk, Septoria infested grains were distributed on each plot at BBCH 31/32 at a density of 25 g/m^2^. Visual assessment of first and flag leaf was performed 32 and 48 days after inoculation^38^. Low values indicate low infection and vice versa.

#### Resistance to Tan spot (DTR)

Inoculations were made with naturally infected straw, stubble and artificially infected grains (prepared by inoculating autoclaved wheat grains with tan spot isolates, viz. a mixture of JKI-Nos. 2009-01, 2009-02 and 2009-07). Before sowing in October, the natural inoculants were soil incorporated at density of 1 kg inoculant/m^2^ of land. To augment infection risk, additional spring inoculation was done wherein fungus infested grains were distributed on each plot at BBCH 21–25 at a density of 25 g/m^2^. Visual assessment of first and flag leaf was performed at BBCH 65–69 (70 days after spring inoculation) and BBCH 83 (90 days after spring inoculation). In total, 10 flag and 10 first leaves were evaluated for each assessment and score for a genotype was calculated as the mean infected area for 10 samples for a given leaf and assessment^39^. Low values indicate low infection and vice versa.

## Technical Validation

### The genotyping arrays deliver complementary data for GABI-WHEAT panel

The marker overlaps between the three arrays (Table 2) are complementary and with the exception of 715 common markers between 35k and 135k chip, little overlap exists between pairs of chips.

**Table 2:** Marker overlaps between different chips.

	35k chip	90k chip	135k chip
**35k chip**	35143	0	715
**90k chip**	0	81588	0
**135k chip**	715	0	136780

### High genetic diversity of the GABI-WHEAT panel is retained with high marker densities and in subset GABI-WHEAT-TROST panel

**Fig. 2:**
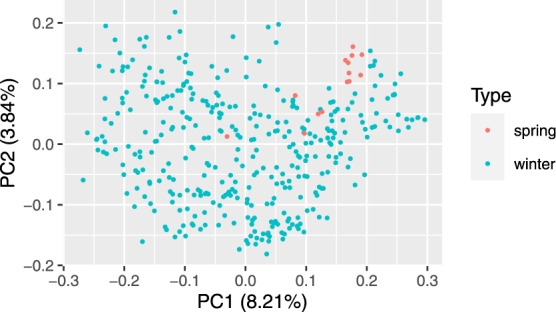
Population structure based on principal coordinate analysis (PCo) using classical multidimensional scaling based on pairwise estimates of Rogers’ distance(s) derived from 90k chip. PC1 and PC2 represent the first two principle components.

**Fig. 3:**
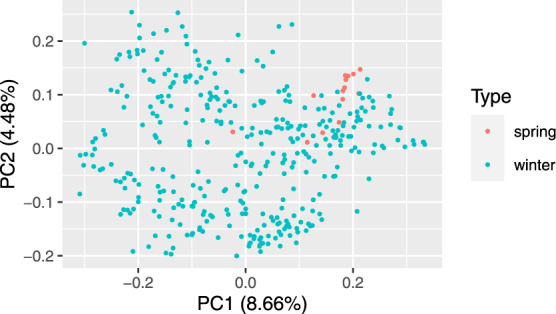
Population structure based on principal coordinate analysis (PCo) using classical multidimensional scaling based on pairwise estimates of Rogers’ distance(s) derived from 35k chip. PC1 and PC2 represent the first two principle components.

**Fig. 4:**
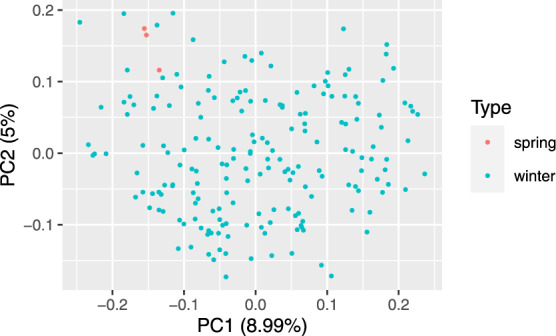
Population structure based on principal coordinate analysis (PCo) using classical multidimensional scaling based on pairwise estimates of Rogers’ distance(s) derived from 135k chip. PC1 and PC2 represent the first two principle components.

**Fig. 5:**
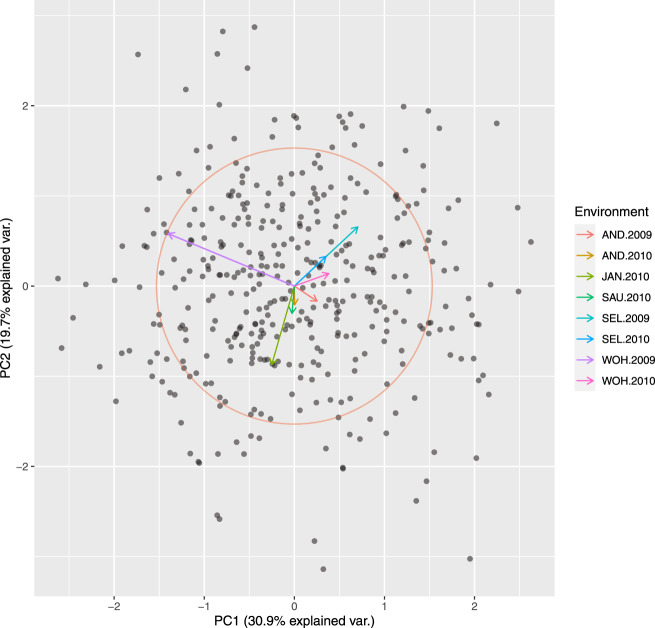
Biplot from rank two approximation of centered g*e matrix for grain yield.

**Fig. 6:**
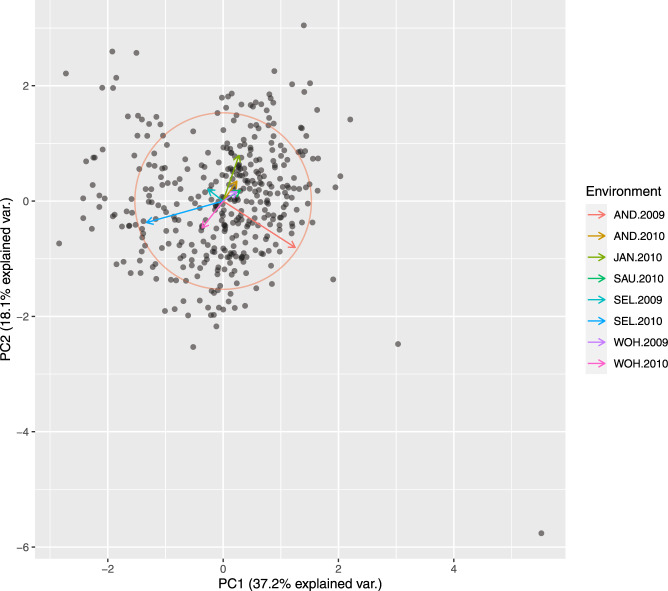
Biplot from rank two approximation of centered g*e matrix for heading date.

**Fig. 7:**
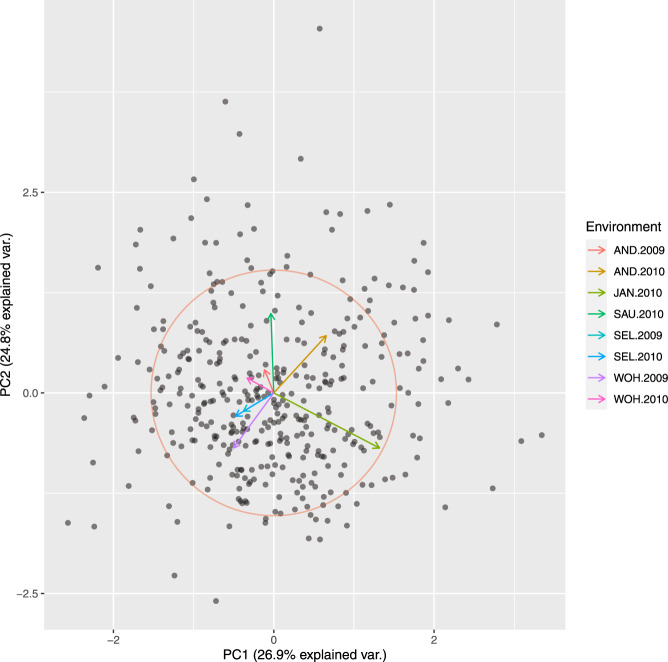
Biplot from rank two approximation of centered g*e matrix for plant height.

**Fig. 8:**
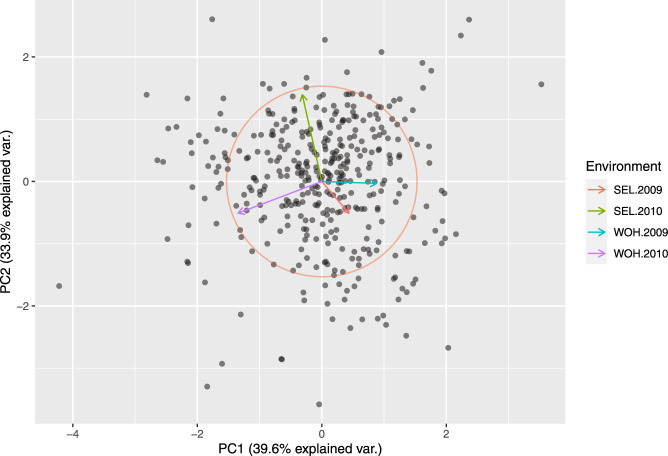
Biplot from rank two approximation of centered g*e matrix for hagberg falling number.

**Fig. 9:**
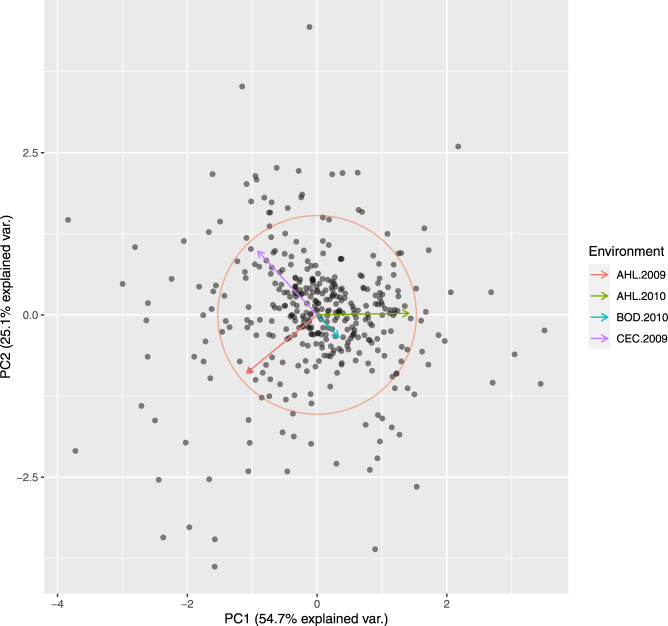
Biplot from rank two approximation of centered g*e matrix for fusarium head blight.

Principle coordinate analysis based on pairwise Rogers’ distance matrix of 371 genotypes using 90k data (Fig. 2), 372 genotypes using 35k (Fig. 3) and the subset of 186 genotypes using 135k data (Fig. 4) agree with past reports^4^ and shows no trend whatsoever across winter or spring wheat genotypes. For traits with complete and balanced data i.e. YIE, HD, PH, HAG and FHB (Figs. 5 to 9), a biplot analysis similar to the principle coordinate analysis revealed (1) no clustering for genotypes for the respective traits (2) no patterns of clustering for environments across the traits. Clustering of environments for any specific trait as well as outlier genotypes for particular environments, were however discovered and may be identified in the interactive plot provided in additional data^29^.

### The distribution of BLUEs approaches normality for majority of the traits

**Fig. 10:**
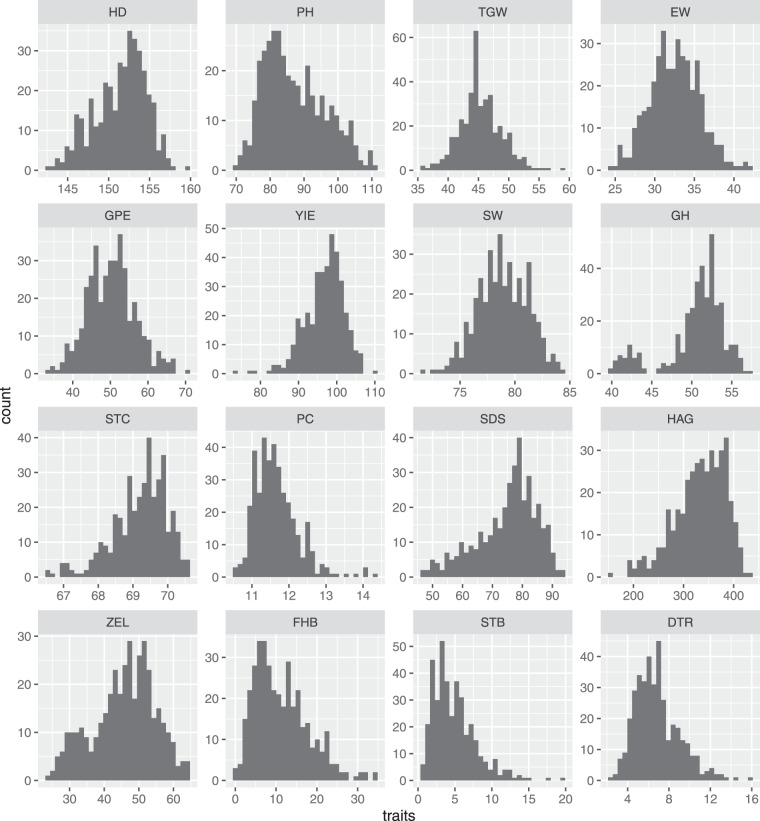
Plot showing distribution of BLUEs for agronomic, quality and disease traits.

Raw data was adjusted for design effects to derive best linear unbiased estimates (BLUEs) across environments for all traits^29^. The BLUEs for most agronomic traits (Fig. 10) were normally distributed except for HD as well as PH, which had slight left and right skew(s), respectively, and for GPE which had a bimodal distribution. Similarly, for quality traits, GH showed a minor secondary peak towards the left end of the distribution, PC was slightly rightly skewed, and others like SDS, STC as well as HAG showed slight left skew. Interestingly however, all disease traits showed a right skew implying only a few of the genotypes were highly susceptible for a given disease. Further, Shapiro–Wilk test for normality revealed that BLUEs for all traits all except SW (pval = 0.27), EW (pval = 0.52) and GPE (pval = 0.07) were normally distributed.

**Fig. 11:**
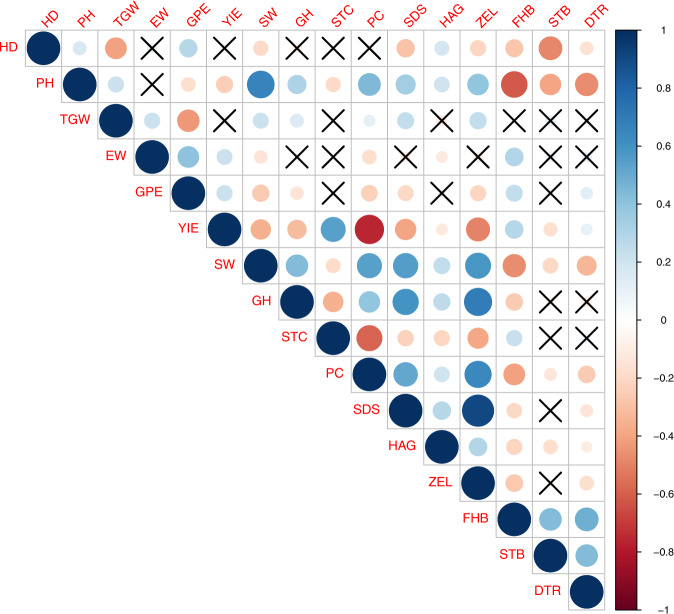
Correlation plot showing significant correlations between BLUEs of agronomic, quality and disease traits. Crosses imply non-significant correlations.

Several significant correlations were observed between BLUEs of traits (Fig. 11) both within and across the broad grouping of agronomic, quality and disease traits. Whereas, within agronomic traits majority pairings except those of GPE, HD with YIE and GPE, YIE with PH showed significant positive correlations, only STC showed negative correlations with all others within quality traits. Interestingly, all pairings within disease traits showed positive correlations. Across the three broad groups, disease traits were predominantly negatively correlated with agronomic and quality traits, except for pairings of EW, GPE, YIE, as well as STC with FHB and those of GPE as well as YIE with DTR respectively. For pairings of agronomic and quality traits it was observed that PH was positively correlated with all quality traits except STC and TKW was positively correlated to majority of quality traits excepting HAG. Other possible pairings of agronomic and quality traits were majorly negatively correlated with each other, barring those of HAG with HD and YIE with SDS.

### Heritability estimates are high for traits phenotyped in multiple environments

**Fig. 12:**
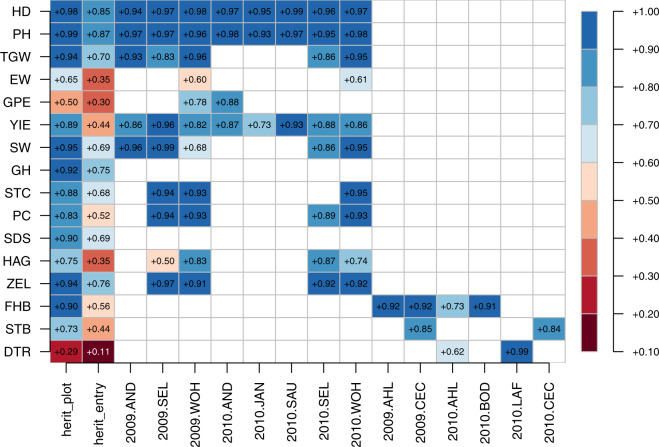
Heatplot showing heritability (column 1 and 2) and repeatability estimates (column 3 onwards) for agronomic, disease and quality traits in the respective environments.

The traits considered in the study can be clustered into three broad groups based on number of replications present per environment wherein they were phenotyped into 1. Those with 2 or more replications per environment (EW, HD, PH, YIE, HAG, STC, SW, FHB, SEP, DTR), 2. Those with one replication per environment (SDS, GH) and 3. Those with upto two replications at a given environment (GPE, TKW, PC, ZEL). Repeatabilities were evaluated for those environments which had at least two replications and the estimates thereof for respective traits suggests high quality of phenotypic data (Fig. 12). The same trend continues for plot mean based heritabilities wherein, excepting traits which were phenotyped in upto three environments (GPE, EW, DTR, SEP), the estimates are high (Fig. 12). Expectedly, the estimates are in line with previous works for GH, PH, HD, TGW, TW, EW^34^, and STC^7^ respectively. Entry mean based heritabilities were at par or in most cases higher than plot based heritabilities.

### The fit of genomic data to BLUEs of respective traits improves with modelling additive*additive epistasis

**Fig. 13:**
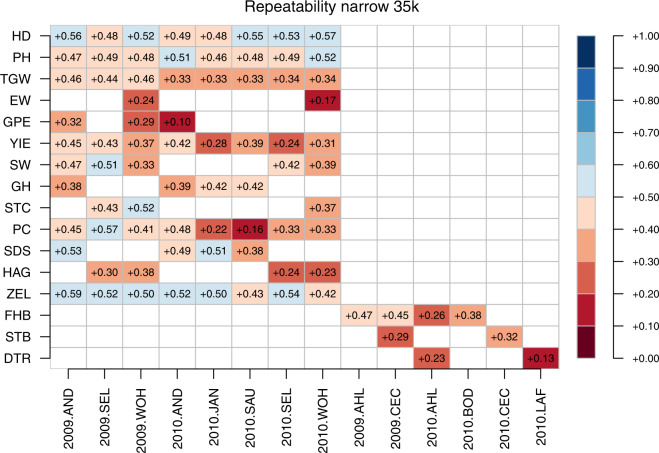
Narrow sense genomic repeatabilities for respective combination of environments and traits based on 35k chip.

**Fig. 14:**
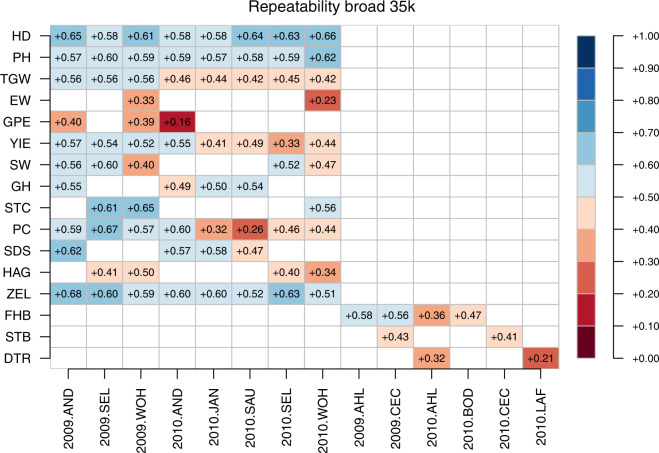
Broad sense genomic repeatabilities for respective combination of environments and traits based on 35k chip.

**Fig. 15:**
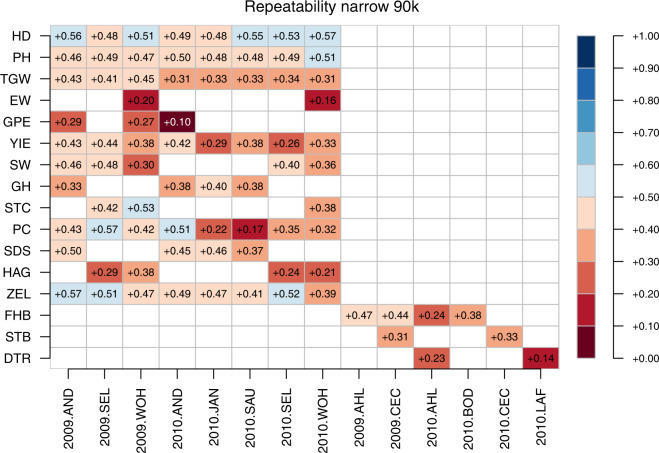
Narrow sense genomic repeatabilities for respective combination of environments and traits based on 90k chip.

**Fig. 16:**
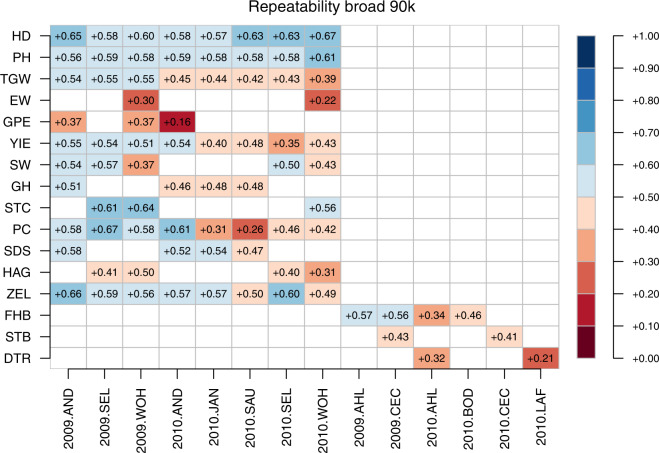
Broad sense genomic repeatabilities for respective combination of environments and traits based on 90k chip.

**Fig. 17:**
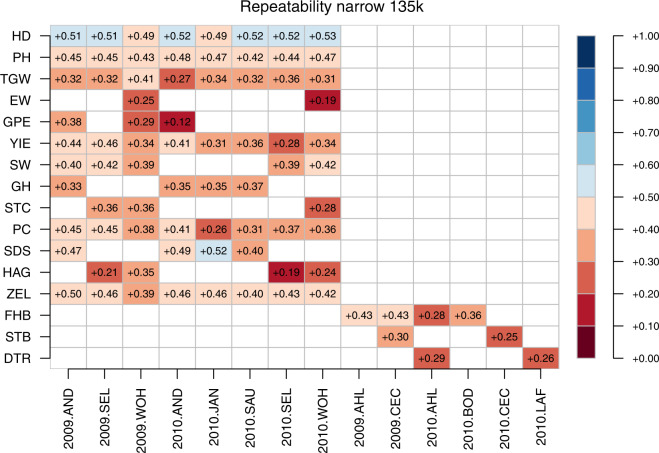
Narrow sense genomic repeatabilities for respective combination of environments and traits based on 135k chip.

**Fig. 18:**
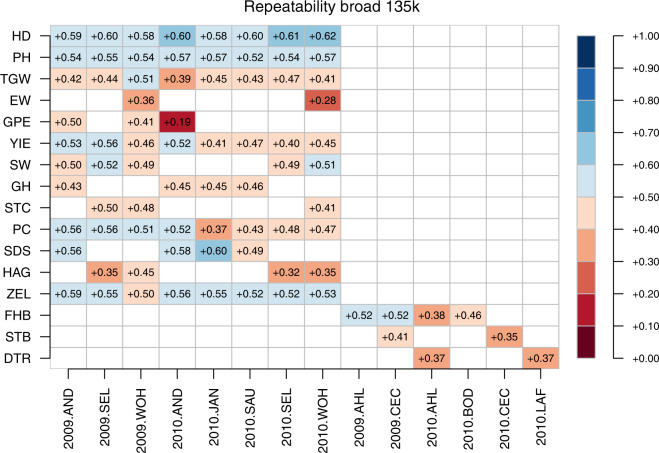
Broad sense genomic repeatabilities for respective combination of environments and traits based on 135k chip.

The three marker datasets reported herein were assessed for their fit to 1. BLUEs within environments and 2. BLUEs across environments by estimating their respective broad sense and narrow sense genomic repeatabilities. The estimates of broad sense genomic repeatabilities were consistently higher for a given combination of trait and environment compared to corresponding narrow sense heritabilities (Figs. 13 to 18). The higher estimates of the former not only highlight the advantage of modelling epistasis for predicting line performance.

### High genomic prediction accuracies support the interoperability of genomic and phenotypic data

**Fig. 19:**
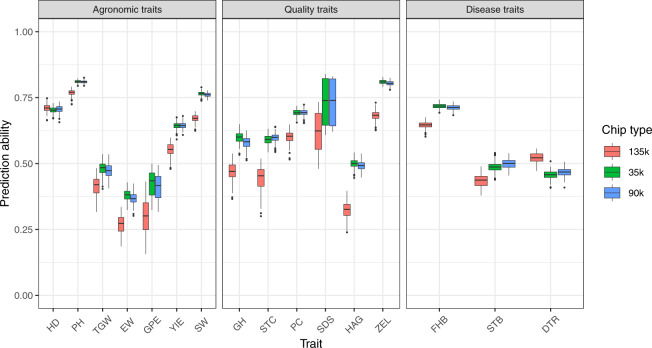
Boxplots showing distributions of 5-fold cross validation runs (100x) for the three marker platforms for respective agronomic, quality and disease traits.

The varying marker densities used to predict respective traits herein reveal counter-intuitive results wherein, markers derived from 35k and 90k chip perform at par (Fig. 19). The prediction abilities with markers derived from 135k chip for most phenotypes are in most cases lower compared to those derived from other chips since the number of genotypes is almost halved with the 135k chip. Interestingly however, the higher marker density of 135k chip yields close results to the other for disease traits and surpasses the other two for DTR. The redundancy observed stems from robust fit of used model in assessing genotype performance(s) for a given trait. The higher marker density however has uses in GWAS augmented with precision phenotyping for instance^40^.

## Data Availability

Preliminary script for processing ISA-TAB files and estimating BLUEs is accessible at https://github.com/AbhishekGogna/GABI-WHEAT/tree/master/output_data.
